# A prospective national cohort study of patients with secondary spontaneous pneumothorax assessing the impact of frailty at diagnosis on mortality and admission to hospital

**DOI:** 10.1183/23120541.00316-2025

**Published:** 2025-12-01

**Authors:** Eleanor C. Barton, Roxanna Short, Alessia Verduri, Jonathan Hewitt, Steven Walker, Ben Carter, Nick A. Maskell

**Affiliations:** 1 Academic Respiratory Unit, School of Clinical Sciences, University of Bristol, Bristol, UK; 2 Department of Biostatistics and Health Informatics, Social Genetic and Developmental Psychiatry Centre, Institute of Psychiatry, Psychology and Neuroscience, King's College London, London, UK; 3 Respiratory Unit, Department of Surgical and Medical Sciences, University of Modena and Reggio Emilia, Hospital Policlinico, Modena, Italy; 4 Division of Population Medicine, School of Medicine, Cardiff University Cardiff, UK

## Abstract

**Aim:**

Secondary spontaneous pneumothorax (SSP) most commonly occurs in older patients with known underlying lung disease. Many are frail, but the effect of frailty on outcomes has not been explored previously. This study aims to evaluate the association between frailty and healthcare outcomes in patients with SSP.

**Methods:**

Patients with SSP were identified from the national Secure Anonymised Information Linkage databank. Frailty status was assessed using the electronic frailty index. The primary outcome was time from diagnosis to all-cause mortality. Secondary outcomes included time from diagnosis to disease-specific mortality and admission to hospital. Data were analysed using a multilevel Cox proportional hazards regression model, adjusted for age, sex, Welsh Index of Multiple Deprivation, smoking status and comorbidities.

**Results:**

Our search identified 3535 individuals diagnosed with SSP between 1 January 2005 and 1 March 2023. By the end of the study, 2102 (59.6%) participants had died with a median follow-up of 683 days (interquartile range 159–1650 days). There was an increasing risk of mortality for those with mild (adjusted hazard ratio (aHR) 1.24, 95% CI 1.10–1.39), moderate (aHR 1.46, 95% CI 1.25–1.70) and severe (aHR 1.83, 95% CI 1.43–2.32) frailty compared to fit individuals. There was also an association between frailty and time to first all-cause hospitalisation, but not disease-specific hospitalisation.

**Conclusions:**

Frailty status at diagnosis was an independent predictor of all-cause mortality in patients with SSP. This demonstrates the importance of assessing frailty status to enable clinicians to provide optimised care and make informed decisions about management of patients with SSP.

## Shareable abstract

**Frailty status at diagnosis is an independent predictor of all-cause mortality in patients with SSP. It is important to assess frailty status to enable clinicians to provide optimised care and make informed decisions about management of patients with SSP.** https://bit.ly/3Gx8VH9

## Introduction

The global population is ageing rapidly; by 2050, there will be an estimated 1.5 billion people worldwide aged >65 years, many of these living with frailty [1]. Frailty is a well-recognised syndrome, describing decreased reserve and an increased vulnerability to external stressors as a result of gradual decline in multiple physiological systems associated with ageing [2, 3]. Although frailty is more common among the elderly, frailty and old age are not synonymous, with frailty describing a comprehensive, multidimensional assessment of the patient and their susceptibility to insults more nuanced than assessing on age alone.

Frailty has been demonstrated to correlate with increased risk of mortality, hospitalisation and long-term institutionalisation almost universally across medical and surgical specialties [4–8]. It is therefore important that clinicians have an understanding both of the tools available to assess frailty and the relationship between frailty and outcomes. Numerous assessment methods have been developed to assess frailty, and generally utilise one of two models: the phenotype model, in which three or more out of five criteria must be met to be defined as frail [3]; or frailty indices, in which accumulated deficits across multiple domains are assessed and used to make a comprehensive, quantifiable assessment of a patient's frailty status [8].

Frailty, its assessment, and clinicians’ understanding of the impact frailty may have on patients is under-recognised and under-represented in respiratory medicine, despite frailty being common among those with respiratory disease. Until recently, the majority of research into frailty and respiratory disease has focussed on malignant disease, COPD and interstitial lung disease (ILD) [9–11]. A recent European Respiratory Society taskforce was developed to summarise existing evidence on frailty in respiratory disease and identify directions for future research, highlighting recognition among respiratory specialists of the importance of promoting frailty assessment in this cohort [12]. Although recent research has begun to explore the relationship between frailty and outcomes in pleural disease [13, 14], frailty in spontaneous pneumothorax remains unexplored.

Spontaneous pneumothorax is the abnormal presence of air in the pleural space, due to a leak of air from the lung itself. It is further classified into primary spontaneous pneumothorax (PSP), occurring in those with presumed normal underlying lung parenchyma, and secondary spontaneous pneumothorax (SSP), occurring in patients with known underlying lung disease or in those aged ≥50 years with a smoking history [15]. SSP is more common than PSP, representing ∼60% of the spontaneous pneumothorax cohort, and is associated with higher rates of recurrence [16]. Patients with SSP are generally older, frailer and more comorbid than PSP patients; COPD and emphysema are generally considered the commonest underlying diseases associated with SSP [17], both of which confer a significant burden of morbidity and mortality. In addition, SSPs can be more refractory to medical management, with longer time to resolution and length of hospital stay and higher mortality rates documented in those with SSP than PSP [18–20]. Treatment options, particularly surgical intervention, are often limited due to patients’ poor functional baseline, despite robust evidence to demonstrate that conservative measures to reduce recurrence rates are inferior to surgical intervention [19, 21, 22].

Despite the widespread use of performance status in clinical practice to guide management in SSP [21, 23], there is limited evidence exploring the relationship between frailty and outcomes in SSP. Many of the largest studies of SSP to date have not reported frailty status [16, 24] although an association between older patients (>70 years) and increased risk of death following spontaneous pneumothorax has been reported. However, this study did not consider frailty [25]. Small, single-centre retrospective studies have shown higher post-operative complications in those with performance status ≥3 and age >80 years, but none have made comprehensive assessments of frailty in surgical cohorts [21, 26].

The Secure Anonymised Information Linkage (SAIL) Databank is a large government-funded national dataset based in Swansea (Wales), containing data for almost 90% of Welsh population. It utilises read codes for cumulative deficits associated with frailty from a patient's electronic linked health records to calculate an established and validated frailty score, the electronic frailty index (eFI) [5]. In this study, we use the SAIL Databank to identify patients with secondary spontaneous pneumothorax, stratify them according to their frailty status and explore the relationship between frailty and outcomes, including mortality, length of stay and readmissions at a population level. This is the first study to explore the relationship between frailty and healthcare outcomes in patients with SSP.

## Methods

### Study design

This study's protocol was published on Research Square on 6 May 2024 [27]. The statistical analysis plan was drafted by a medical statistician fully blinded to the outcome data (B. Carter) in accordance with King's College London Clinical Trials Unit standard operating procedure on drafting a statistical analysis plan. The study was funded by Cardiff University's Wellcome Trust Institutional Translation Partnership Award (Access to Expertise award, identifier number 214601/Z/18/Z) and a discretionary award from the Academic Respiratory Unit, University of Bristol (Bristol, UK).

### Data sources

We used the SAIL Databank, a large national dataset containing health records of ∼90% of Wales’ 3 million population [28]. SAIL comprises multiple linked datasets including the Welsh Longitudinal General Practice Dataset, Patient Episode Dataset for Wales, Outpatient Database for Wales, Emergency Department Data Set, Welsh Demographic Service Dataset – demographics and Welsh Index of Multiple Deprivation (WIMD) decile, and Annual District Death Extract. Information governance review panel approval was obtained to access the data.

### Study participants

Patients with a first episode of spontaneous pneumothorax between 1 January 2005 and 1 March 2023 were extracted from the databank using the International Classification of Diseases 10th revision code J93 (pneumothorax) or National Health Service read code H52 (pneumothorax). Patients with SSP were then selected using the following inclusion criteria: 1) current or ex-smoker aged ≥50 years; or 2) aged ≥18 years with a history of COPD, ILD or thoracic malignancy. Patients were excluded if they met any of the following exclusion criteria: 1) aged <18 years; 2) PSP (as defined by not meeting either of the above inclusion criteria); or 3) aged ≥18 years with missing data.

### Primary outcome

The primary outcome was time to all-cause mortality from diagnosis with SSP. Participants were censored at the time last known to be alive. Those who did not have a date of death at the end of the study period (1 March 2023) were assumed alive and censored at that time point.

### Secondary outcomes

Secondary outcomes were time to disease-specific mortality, time to first all-cause and disease-specific hospital admission, length of stay of first all-cause and disease-specific hospital admissions after the diagnosis of spontaneous pneumothorax and any readmission within 90 days of first all-cause and disease-specific admission. Hospital admissions were defined as an attendance to hospital lasting ≥24 h and in which the patient did not die on the day of admission. Patients were censored at this time point if death occurred on the day of hospital admission.

### Exposures

Patients’ frailty was assessed at the date of diagnosis with spontaneous pneumothorax using the eFI [5], calculated within the SAIL Databank. Patients were classified into categories of frailty: fit (0–0.12), mildly frail (>0.12–0.24), moderately frail (>0.24–0.36) or severely frail (>0.36) based on their eFI score. Baseline covariates collected included age at presentation, sex, WIMD and comorbidities, as assessed using the Charlson Comorbidity Index and the presence of specific comorbidities including COPD and congestive heart failure (CHF).

### Data analysis

The impact of frailty status (fit, mild, moderate, severe) on time to all-cause mortality was assessed using multilevel Cox proportional hazards regression, adjusting for age (<50, 50–64, 65–74, 75–84 and ≥85 years), sex (female, male), WIMD quintile (Q1–Q5), smoking status (never, ex-, current), Charlson Comorbidity Index (0–≥5), presence of COPD or CHF (yes/no). Crude and adjusted hazard ratios (aHR) were reported, with associated p-values and 95% confidence intervals. A baseline proportional hazards assumption model was assessed visually using log–log plots.

Secondary outcomes were analysed using a time-to-event analysis consistent with the primary outcome. The impact of frailty status on 90-day all-cause and disease-specific readmission were analysed using a multilevel logistic regression model, adjusting for the same covariates as the primary outcome.

## Results

**FIGURE 1:**
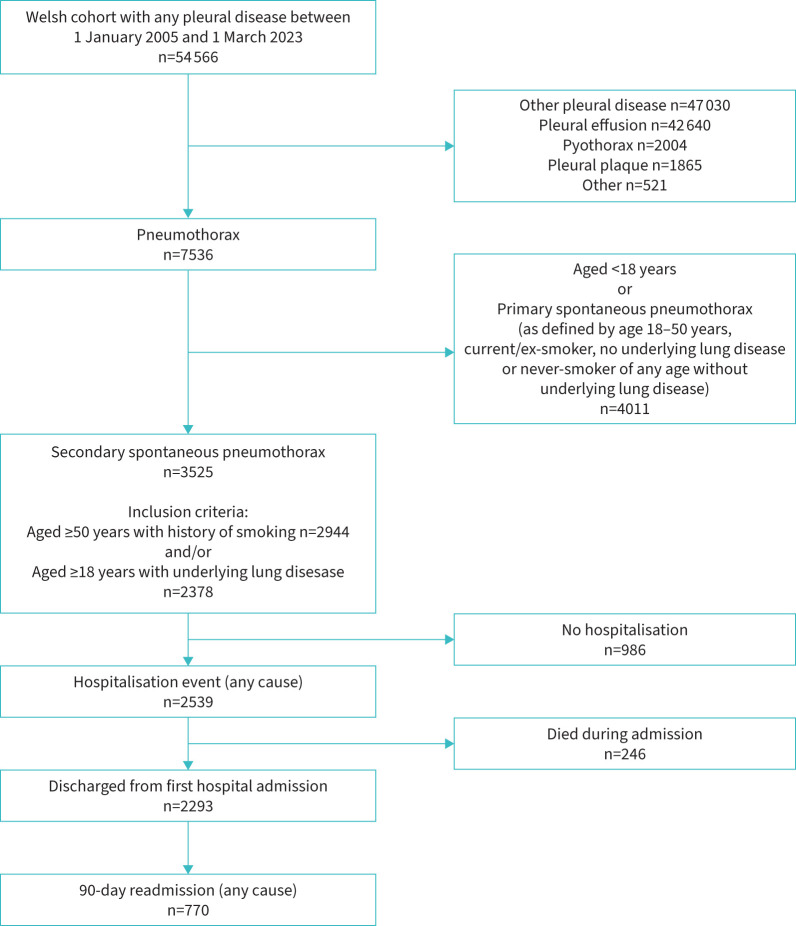
Consolidated Standards of Reporting Trials diagram showing the cohort size for each analysis.

The data were first accessed on 27 July 2023. The final sample comprised 3525 individuals who had been diagnosed with SSP between 1 January 2005 and 1 March 2023 (figure 1) Median (interquartile range (IQR) age was 69 (60–77) years; 1212 (34.4%) participants were female and 2313 (65.6%) were male. 2944 patients were classified as SSP, as they were aged ≥50 years with a history of smoking; 2378 patients were patients with underlying lung diseases (COPD, ILD, lung cancer or mesothelioma, or pulmonary hypertension). 3111 (88.2%) participants were current or former smokers. In terms of missing data, two (<0.1%) participants had missing deprivation indices at the start of the study period.

By the end of the study period, 2102 (59.6%) participants had died, with a median (IQR) follow-up of 683 (159–1650) days. The median (IQR) follow-up for those who survived was 2017 (905–3664) days.

**FIGURE 2:**
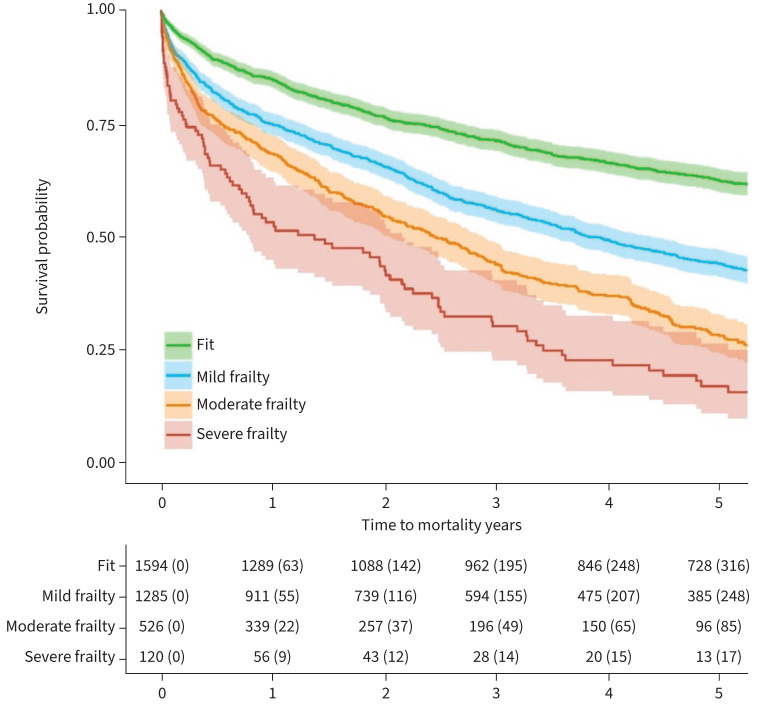
Kaplan–Meier plot showing overall survival by electronic frailty index categories (n=3525).

Cause of death was missing for the majority of the sample, and was therefore could not be analysed. 1594 (45.2%) participants were classified as fit on the eFI, with 1285 (36.5%) classified as mildly frail, 526 (14.9%) as moderately frail and 120 (3.4%) as severely frail (table 1). The median survival for those classified as without frailty (*i.e.* classified as fit) was 8.38 (95% CI 7.77–9.59) years compared with 3.82 (95% CI 3.82–4.38) years for those with mild frailty, 2.43 (2.05–28.7) years for those with moderate frailty, and 1.37 (0.79–2.16) years for those with severe frailty (figure 2). 819 (51.4%) out of 1594 without frailty (*i.e.* classified as fit) remained alive at the end of follow-up. 462 (36.0%) out of 1285 with mild frailty, 120 (22.8%) out of 526 with moderate frailty, and 22 (18.3%) out of 120 with severe frailty remained alive at the end of follow-up. The 1-year mortality rates for those classified as fit, mildly frail, moderately frail and severely frail were 0.15, 0.25, 0.31 and 0.46, respectively.

**TABLE 1:** Population characteristics at baseline by mortality status (n=3520)

	Alive	Dead	Total
**Frailty**			
Fit	819 (57.6)	775 (36.9)	1594 (45.2)
Mild	462 (32.5)	823 (39.2)	1285 (36.5)
Moderate	120 (8.4)	406 (19.3)	526 (14.9)
Severe	22 (1.5)	98 (4.7)	120 (3.4)
**Age years**			
<65	773 (54.3)	525 (25.0)	1298 (36.8)
65–74	406 (28.5)	711 (33.8)	1117 (31.7)
75–84	199 (14.0)	646 (30.7)	845 (24.0)
≥85	45 (3.2)	220 (10.5)	265 (7.5)
**Sex**			
Male	914 (64.2)	1399 (66.6)	2313 (65.6)
Female	509 (35.8)	703 (33.4)	1212 (34.4)
**WIMD quintile**			
1	343 (24.1)	539 (25.6)	882 (25.0)
2	315 (22.1)	501 (23.8)	816 (23.1)
3	315 (22.1)	481 (22.9)	796 (22.6)
4	258 (18.1)	312 (14.8)	570 (16.2)
5	191 (13.4)	268 (12.7)	459 (13.0)
Missing	1 (0.1)	1 (0.0)	2 (0.1)
**COPD**			
No	787 (55.3)	874 (41.6)	1661 (47.1)
Yes	636 (44.7)	1228 (58.4)	1864 (52.9)
**Heart failure**			
No	1353 (95.1)	1935 (92.1)	3288 (93.3)
Yes	70 (4.9)	167 (7.9)	237 (6.7)
**Charlson Comorbidity Index**			
0	357 (25.1)	280 (13.3)	637 (18.1)
1	121 (8.5)	127 (6.5)	258 (7.3)
2	62 (4.4)	77 (3.7)	139 (3.9)
3	270 (19.0)	334 (15.9)	604 (17.1)
4	212 (14.9)	342 (16.3)	554 (15.7)
≥5	401 (28.2)	932 (44.3)	1333 (37.8)
**Smoking status**			
Current	375 (26.4)	473 (22.5)	848 (24.1)
Former	947 (66.5)	1316 (62.6)	2263 (64.2)
Never	101 (7.1)	313 (14.9)	414 (11.7)

Data are presented as n (%). WIMD: Welsh Index of Multiple Deprivation.

### Primary outcome

There was an association between frailty and all-cause mortality; compared with fit individuals, there was an increasing risk of mortality for those with mild, moderate and severe frailty (table 2; figure 2). Age appeared to be a key confounder of this association, but the direction of the effects of frailty remained consistent before and after adjustment. Being in WIMD quintile 1 or 2 and having any comorbidities on the Charlson Comorbidity Index were also associated with increased mortality. Female sex or having a diagnosis of heart failure was associated with reduced mortality (table 2).

**TABLE 2:** Time to all-cause mortality (n=3520^#^; n=2096 events)

	HR (95% CI)	p-value	aHR (95% CI)	p-value
**Frailty**				
Fit	1 (ref.)		1 (ref.)	
Mild	1.76 (1.60–1.95)	<0.001	1.24 (1.10–1.39)	<0.001
Moderate	2.73 (2.41–3.09)	<0.001	1.46 (1.25–1.70)	<0.001
Severe	3.85 (3.11–4.77)	<0.001	1.83 (1.43–2.32)	<0.001
**Age years**				
<65	1 (ref.)		1 (ref.)	
65–74	2.45 (2.18–2.75)	<0.001	2.25 (1.99–2.53)	<0.001
75–84	3.68 (3.26–4.14)	<0.001	3.11 (2.73–3.54)	<0.001
≥85	5.64 (4.79–6.64)	<0.001	4.36 (3.64–5.22)	<0.001
**Sex**				
Male	1 (ref.)		1 (ref.)	
Female	0.99 (0.90–1.08)	0.780	0.90 (0.82–0.98)	0.019
**WIMD quintile**				
1	1.05 (0.90–1.21)	0.547	1.28 (1.10–1.49)	0.001
2	1.10 (0.94–1.27)	0.223	1.22 (1.05–1.42)	0.009
3	1.04 (0.90–1.21)	0.584	1.15 (0.99–1.34)	0.069
4	0.92 (0.78–1.09)	0.345	0.93 (0.79–1.10)	0.416
5	1 (ref.)		1 (ref.)	
**COPD**				
No	1 (ref.)		1 (ref.)	
Yes	1.07 (0.98–1.17)	0.141	0.99 (0.90–1.08)	0.771
**Heart failure**				
No	1 (ref.)		1 (ref.)	
Yes	1.11 (0.94–1.30)	0.208	0.75 (0.64–0.88)	<0.001
**Charlson Comorbidity Index**				
0	1 (ref.)		1 (ref.)	
1	1.74 (1.41–2.13)	<0.001	1.39 (1.13–1.72)	0.002
2	1.85 (1.43–2.38)	<0.001	1.38 (1.06–1.79)	0.016
3	1.35 (1.15–1.58)	<0.001	1.18 (1.00–1.39)	0.051
4	1.90 (1.62–2.23)	<0.001	1.37 (1.15–1.63)	<0.001
≥5	2.59 (2.26–2.97)	<0.001	1.46 (1.24–1.72)	<0.001

(a)HR: (adjusted) hazard ratio (adjusted for baseline covariates); WIMD: Welsh Index of Multiple Deprivation; ref.: reference. ^#^: n=5 died on date of diagnosis, n=2 had missing WIMD.

### Secondary outcomes

There was an increasing association with worsening frailty and time to first all-cause hospitalisation. Compared with fit individuals, there was an increasing risk of hospitalisation for those with mild, moderate and severe frailty (table 3). Increasing age, female sex, a diagnosis of COPD and the presence of any comorbidities on the Charlson Comorbidity Index were associated with increased risk of all-cause hospitalisation. However, frailty was not associated with pneumothorax-related hospital admission. Conversely, female sex conferred a reduced risk of disease-specific hospitalisation. There was no association between frailty and length of any-cause hospital admission nor between frailty and 90-day any-cause or pneumothorax-related hospital readmission. We were unable to assess the association between frailty and disease-specific mortality due to missing data regarding cause of death.

**TABLE 3:** Time to any-cause hospitalisation (n=3520^#^; n=2537 events)

	HR (95% CI)	p-value	aHR (95% CI)	p-value
**Frailty**				
Fit	1 (ref.)		1 (ref.)	
Mild	1.47 (1.35–1.61)	<0.001	1.21 (1.09–1.34)	<0.001
Moderate	1.80 (1.60–2.02)	<0.001	1.32 (1.15–1.53)	<0.001
Severe	2.17 (1.73–2.72)	<0.001	1.45 (1.13–1.87)	0.004
**Age years**				
<65	1 (ref.)		1 (ref.)	
65–74	1.39 (1.26–1.53)	<0.001	1.24 (1.13–1.37)	<0.001
75–84	1.68 (1.51–1.87)	<0.001	1.40 (1.25–1.57)	<0.001
≥85	1.80 (1.52–2.12)	<0.001	1.44 (1.21–1.72)	<0.001
**Sex**				
Male	1 (ref.)		1 (ref.)	
Female	1.15 (1.06–1.25)	<0.001	1.09 (1.01–1.19)	0.037
**WIMD quintile** ^¶^				
1	1.10 (0.96–1.26)	0.161	1.12 (0.98–1.29)	0.094
2	1.11 (0.97–1.28)	0.127	1.10 (0.96–1.27)	0.162
3	1.08 (0.94–1.24)	0.278	1.09 (0.95–1.25)	0.236
4	1.03 (0.89–1.20)	0.651	1.03 (0.89–1.20)	0.682
5	1 (ref.)		1 (ref.)	
**COPD**				
No	1 (ref.)		1 (ref.)	
Yes	1.29 (1.19–1.40)	<0.001	1.20 (1.10–1.30)	<0.001
**Heart failure**				
No	1 (ref.)		1 (ref.)	
Yes	1.36 (1.18–1.57)	<0.001	1.12 (0.96–1.30)	0.143
**Charlson Comorbidity Index**				
0	1 (ref.)		1 (ref.)	
1	1.36 (1.14–1.63)	<0.001	1.29 (1.08–1.54)	0.006
2	1.50 (1.19–1.88)	<0.001	1.32 (1.05–1.67)	0.018
3	1.34 (1.17–1.52)	<0.001	1.15 (1.00–1.32)	0.044
4	1.48 (1.29–1.70)	<0.001	1.18 (1.02–1.38)	0.026
≥5	1.82 (1.62–2.05)	<0.001	1.29 (1.12–1.48)	<0.001

(a)HR: (adjusted) hazard ratio (adjusted for baseline covariates); WIMD: Welsh Index of Multiple Deprivation; ref.: reference. ^#^: n=5 died on date of diagnosis, n=2 had missing WIMD.

### Subgroup analyses

In our subgroup analyses, compared to fit individuals, frailty (whether mild, moderate or severe) was associated with increased risk of mortality in all subgroups, with the exception of female gender (table 4). There was a stronger association between frailty and mortality in those aged <70 years, compared to those aged ≥70 years, in males compared to females, in those with a diagnosis of COPD compared to those without and in those who had never smoked compared to current/former smokers.

**TABLE 4:** Subgroup analyses of the association between frailty (electronic frailty index >0.12) and mortality in secondary spontaneous pneumothorax by frailty status

	aHR (95% CI)	p-value
**Age**		
Frail + <70 years	1.57 (1.34–1.85)	<0.001
Frail + ≥70 years	1.23 (1.06–1.42)	0.008
**Sex**		
Frail + male	1.60 (1.40–1.85)	<0.001
Frail + female	1.10 (0.92–1.33)	0.295
**COPD status**		
Frail + COPD	1.27 (1.08–1.50)	0.005
Frail + no COPD	1.50 (1.29–1.74)	<0.001
**Smoking status**		
Frail + never-smoker	1.44 (1.27–1.62)	<0.001
Frail + former/current smoker	1.29 (1.03–1.62)	0.027

Fit individuals in each subgroup (*e.g.* age <70 years/≥70 years) were used as reference cohorts in this analysis. (a)HR: (adjusted) hazard ratio (adjusted for baseline covariates).

## Discussion

In this national cohort study of 3525 patients with SSP, we found that increased frailty at diagnosis was linked to increased all-cause mortality and faster all-cause hospitalisation. SSP patients with severe frailty had a high risk of 1-year mortality.

This is the first study to explore the association between frailty and multiple healthcare outcomes, including mortality, hospital admission, length of stay and readmission, in patients with SSP. Other studies have also demonstrated a link between frailty and poorer healthcare outcomes in trauma [29], surgery [30] and other respiratory conditions, including ILD [10], COPD [9] and pleural disease as a whole [14, 31]; findings that were corroborated by our study.

Prior studies exploring healthcare outcomes in SSP have been limited to small, single-centre, retrospective studies [21, 26], and neither these nor larger studies of the SSP population as a whole have reported frailty status in their cohorts [16, 24]. A previous study identified an increased risk of death in patients with spontaneous pneumothorax aged >80 years [25], a finding also reflected in our data; however, this was a retrospective study of patients admitted to a single tertiary centre, and made no assessment of the patients’ frailty status. The use of population-level data in our study has provided us with a cohort representative of the SSP population as a whole; rates of SSP were comparable with another large epidemiological study, although this had a slightly higher male preponderance and lower age range, probably skewed by the inclusion of patients with primary pneumothorax [16]. Using the eFI has allowed us to assess frailty robustly with a validated tool, rather than relying on age as a proxy measure of patients’ frailty status.

Frailty was not associated with time to pneumothorax-related hospital admission, length of any-cause hospitalisation or 90-day readmission. The pathophysiology underlying recurrence in spontaneous pneumothorax is not well understood, but it is unlikely that frailty is a direct cause for recurrence of SSP and therefore pneumothorax-related readmission. The absence of an association between frailty status and length of hospital admission was surprising, given that increasing frailty is commonly associated with increased length of stay in the literature [14, 29]. In addition, frailer patients are more likely to be offered conservative or medical management of their pneumothorax, a process which can take considerably longer and is therefore often associated with deconditioning and increased rehabilitation and care needs. It is perhaps this, rather than baseline frailty, that may drive longer hospital admissions.

This study does have limitations; we were unable to report on disease-specific mortality due to a significant amount of missing cause-of-death data. We note that the mortality rate for severely frail patients with SSP is comparable to that of severely frail patients in a recent study of pleural disease as a whole [14]; a cohort likely to contain a significant proportion of patients with metastatic malignant disease, which highlights that pneumothorax may not be as benign a condition as it is sometimes thought. To explore this further, association between frailty status and disease-specific mortality should be assessed.

Furthermore, as with most frailty scores, the eFI is not validated in people aged <65 years, who made up 37.8% of our cohort. We note that 45.2% of patients in our cohort were classified as “fit”, a higher percentage than might be expected in our clinical experience, and seemingly disproportionate to the degree of comorbidity recorded in our study, with 53.5% of the population scoring ≥4 on the Charlson Comorbidity Index. In addition, in our subgroup analysis, there was also a stronger association between frailty and mortality in those aged <70 years when compared to fit individuals than those aged ≥70 years. It was not possible to explore the reasons for this result in this study, but this finding raises a broader question of how and whether frailty should be assessed in younger patients.

These results have demonstrated the dose–response relationship between frailty and mortality in patients with SSP, but further research is needed. The SSP population is very diverse, ranging from smokers with no formal diagnosis of an underlying lung condition to elderly patients with advanced fibrotic lung disease; therefore, exploring the relationship between frailty and healthcare outcomes depending on underlying condition would be of great clinical value. In addition, understanding the role of frailty status in selection for surgical intervention, as well as outcomes after surgery would help clinicians make holistic decisions about suitability of their patients for surgical intervention.

This study aimed to explore the association between frailty and healthcare outcomes in the SSP population. Our results also highlighted the importance of understanding a patients’ degree of frailty, rather than frailty as a binary concept, as outcomes between those who are mildly frail are significantly better than those who are severely frail. Understanding these relationships is of great value to clinicians. The options for management of SSP range from conservative management, in which no intervention is performed and patients are monitored either in the inpatient or outpatient setting until their pneumothorax has resolved or requires intervention, to surgical procedures, which are considerably more invasive and higher risk, but have been shown to be more successful at reducing recurrence [19, 21, 22]. Deciding on an appropriate course of action is often a balance between managing the burden of a patient's symptoms and assessing the risk of more invasive management, both in terms of mortality and their quality of life. Having a tool with which clinicians can assess frailty and understanding what a patient's frailty may indicate, in terms of their risk of mortality, readmission and admission duration, can help clinicians make pragmatic decisions about the most appropriate management strategy and advanced care planning, and improve patients’ access to frailty services both during their inpatient stay and in the community.
